# Heart–Brain Relationship in Stroke

**DOI:** 10.3390/biomedicines9121835

**Published:** 2021-12-04

**Authors:** Roger E. Kelley, Brian P. Kelley

**Affiliations:** 1 Ochsner/LSU Health Sciences Center, Department of Neurology, Shreveport, LA 71130, USA; 2 Division of Cardiology, Department of Internal Medicine, University of North Carolina School of Medicine, Chapel Hill, NC 27514, USA; Brian.Kelley@unchealth.unc.edu

**Keywords:** cerebral infarction, cardioembolic stroke, atrial fibrillation, infectious endocarditis, septic emboli, patent foramen ovale, valvular heart disease, congestive heart failure, mural wall thrombus, atrial thrombus, ischemic cardiomyopathy

## Abstract

The patient presenting with stroke often has cardiac-related risk factors which may be involved in the mechanism of the stroke. The diagnostic assessment is predicated on recognition of this potential relationship. Naturally, an accurate history is of utmost importance in discerning a possible cause and effect relationship. The EKG is obviously an important clue as well as it allows immediate assessment for possible cardiac arrhythmia, such as atrial fibrillation, for possible acute ischemic changes reflective of myocardial ischemia, or there may be indirect factors such as the presence of left ventricular hypertrophy, typically seen with longstanding hypertension, which could be indicative of a hypertensive mechanism for a patient presenting with intracerebral hemorrhage. For all presentations in the emergency room, the vital signs are important. An elevated body temperature in a patient presenting with acute stroke raises concern about possible infective endocarditis. An irregular–irregular pulse is an indicator of atrial fibrillation. A markedly elevated blood pressure is not uncommon in both the acute ischemic and acute hemorrhagic stroke setting. One tends to focus on possible cardioembolic stroke if there is the sudden onset of maximum neurological deficit versus the stepwise progression more characteristic of thrombotic stroke. Because of the more sudden loss of vascular supply with embolic occlusion, seizure or syncope at onset tends to be supportive of this mechanism. Different vascular territory involvement on neuroimaging is also a potential indicator of cardioembolic stroke. Identification of a cardiogenic source of embolus in such a setting certainly elevates this mechanism in the differential. There have been major advances in management of acute cerebrovascular disease in recent decades, such as thrombolytic therapy and endovascular thrombectomy, which have somewhat paralleled the advances made in cardiovascular disease. Unfortunately, the successful limitation of myocardial damage in acute coronary syndrome, with intervention, does not necessarily mirror a similar salutary effect on functional outcome with cerebral infarction. The heart can also affect the brain from a cerebral perfusion standpoint. Transient arrhythmias can result in syncope, while cardiac arrest can result in hypoxic–ischemic encephalopathy. Cardiogenic dementia has been identified as a mechanism of cognitive impairment associated with severe cardiac failure. Structural cardiac abnormalities can also play a role in brain insult, and this can include tumors, such as atrial myxoma, patent foramen ovale, with the potential for paradoxical cerebral embolism, and cardiomyopathies, such as Takotsubo, can be associated with precipitous cardioembolic events.

## 1. Introduction

Cardioembolic stroke is common and is a frequent presentation in the emergency room. It has been estimated that one-quarter to one-third of all ischemic strokes are cardioembolic. In a referral population study [1], the percentage of strokes felt to be cardioembolic increased from 22.8% in the 2002–2005 timeframe to 54.3% in the 2009–2012 timeframe. From a management standpoint, just as “time is brain” for thrombolytic therapy, prevention of recurrent cerebral embolism is also of primary importance. The standard diagnostic approach in the acute ischemic stroke setting is a stat computed tomography (CT) brain scan, EKG and routine blood work including a complete blood count, comprehensive metabolic profile as well as prothrombin time (PT)/international normalization ratio (INR) and activated partial thromboplastin time (aPTT). Vascular imaging along with perfusion imaging is now also part of the standard protocol in order to assess potential eligibility for endovascular thrombectomy.

There are certain features of cardioembolic stroke that help to heighten clinical concern about this possible mechanism. However, despite increasing access to vascular neurology specialists and enhanced early imaging, it has been estimated that up to one-third of ischemic strokes remain in the “cryptogenic” category upon initial diagnostic evaluation [2].

**Figure 1:**
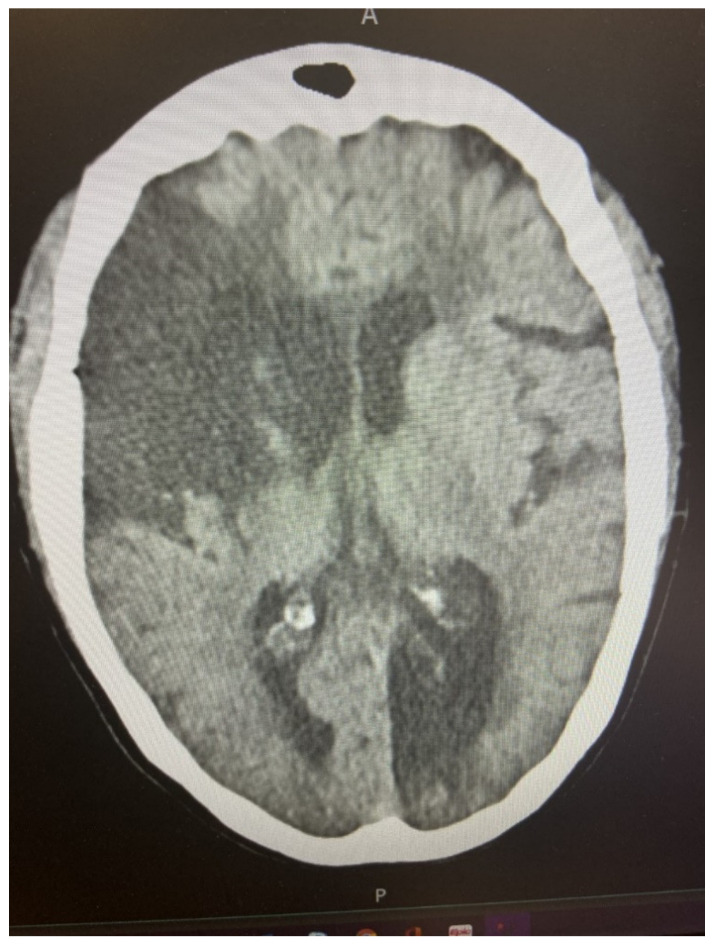
The Potential Effects of Atrial Fibrillation on the Cerebral Circulation. A = anterior, P = posterior.

Supportive features for cardioembolic stroke are summarized in Table 1. A history of atrial fibrillation certainly enhances the likelihood of a cardioembolic mechanism. However, this risk is predicated on coexistent features which will be discussed below. Sudden maximal neurological deficit as opposed to the stepwise or stuttering course associated with thrombotic stroke can be an important clue. It has also been reported that transient ischemic attack (TIA) is more likely a harbinger of thrombotic stroke than cardioembolic stroke, but this is subject to interpretation. For example, one might invoke a “thrombo-embolic” mechanism for certain patients such as a patient with retinal ischemia with ipsilateral, high-grade, carotid stenosis along with Hollenhorst plaque seen on a funduscopic exam. The sudden loss of cerebral blood flow to a discrete vascular territory, related to embolic occlusion, is believed to be more likely to result in associated syncope or seizure with the ictus. The attempt to rapidly restore blood flow to the involved territory, through collateral circulatory channels, is believed to translate into an enhanced risk of hemorrhagic transformation of the infarct. Lack of scattered or diffuse atherosclerotic occlusive disease would tend to support a cardioembolic mechanism along with distinct cutoff of a specific vessel supplying a discrete vascular territory. Multiple vascular territory involvement is particularly supportive of a cardioembolic mechanism (Figure 1).

Atrial fibrillation (AF) is increasingly more common with advancing age. There is a prevalence of roughly 0.1% in subjects <55 years of age while the prevalence approaches almost 10% in subjects >80 years of age [3]. It has been reported that the risk of stroke in the younger patient, <60 years of age, without significant coexistent factors, i.e., “lone atrial fibrillation”, is similar to that of a subject of similar age without AF [4]. This implies a structural normal heart with no identified risk factors for ischemic vascular disease. However, at age 60 and beyond, there is an increasing risk of thromboembolic events [5]. The Framingham study of lone AF emphasized this inherent risk over time, although the mean age was 70.6 years for men and 68.1 years for women in their report [6]. The identification of lone AF being of low risk for cardioembolic events in subjects younger than 60 years translates into the concept of cumulative risk for stroke as the subject ages and accumulates co-morbid factors which enhance stroke risk with AF. This has led to the development of scales to stratify stroke risk associated with AF. For example, the association of AF with rheumatic valvular heart disease is associated with close to an 18-fold enhanced risk of cardioembolic stroke, while nonvalvular AF (NVAF) is associated with a 5-fold increased risk overall according to date from the Framingham study [7]. It is reported that AF is associated with 20 to 30% of all ischemic strokes and 10% of cryptogenic strokes [8]. There is increasing detection of paroxysmal atrial fibrillation with prolonged monitoring of patients presenting with stroke as well as innovative devices such as watches that can preemptively detect this rhythm disturbance. In AF, it is felt that formation of thrombus within the left atrial appendage (LAA) is the primary factor associated with stroke risk [9]. This is generally detected by echocardiography. During AF and atrial flutter, the left atrial appendage loses contractility which can lead to thrombus formation. The reduction in contractility is associated with decreased flow velocities and rouleaux formation of red blood cells. There is a resultant smoke-like signal which can be most readily detected by transesophageal echocardiogram (TEE) and has been termed “spontaneous echo contrast” [10].

There are a number of potential factors which can contribute to enhanced stroke risk with AF. As alluded to above, these include increasing age, valvular heart disease and factors associated with predisposition to thrombus in the LAA. LAA evaluation might allow detection of higher risk patients with AF and potentially serve as an indirect marker of undetected paroxysmal AF. For example, N-terminal probrain natriuretic peptide is a biomarker of atrial integrity and its elevation is associated with increased stroke risk with identified AF [11] as well as risk of cardioembolism in general [12,13]. In terms of thrombus detection within the LAA, TEE is the optimal modality [14]. Of pertinence, in recognition of the more invasive nature of TEE compared to transthoracic echocardiography (TTE), the use of tissue harmonic imaging with TEE [15] has been reported to achieve a detection rate very similar to TEE [16].

The CHA_2_DS_2_-VASc stroke risk score in NVAF represents the most commonly used stratification of risk tool [17]. This is summarized in Table 2 [18]. This widely used scoring system appears to be best suited for lower risk subjects, i.e., males with a score of 0 and females with a score of 1 who do not appear to require any antithrombotic therapy [19]. A number of scores have been developed with the general view that an annual stroke risk of 1 to 2% does not necessarily justify anticoagulant therapy and the additional opinion, among some, that antiplatelet therapy does not necessarily provide significant protection against cardioembolic stroke associated with AF. The annual risk of stroke increases with an increasing CHA_2_DS_2_VASc score, but the reported reliability of the various scales has been questioned [20]. Both The National Institute for Health and Care Excellence (NICE) [21] and the European Society of Cardiology (ESC) [8] have provided guidelines with the recommendation that patients with a CHA_2_DS_2_-VASc score of ≥2 receive oral anticoagulant therapy as long as there are no absolute contraindications to the use of such agents. In prevention of stroke in AF, there are a number of potential approaches including suppression of the AF. There can be electrical cardioversion or cardioversion with drugs. Such cardioversion is typically followed by antiarrhythmic medications, which can include amiodarone, flecainide, propafenone, sotolol or dofetilide. Cardiac ablation is an alternative approach to suppress the atrial fibrillation particularly if an initial attempt at cardioversion is unsuccessful. Either a radiofrequency or cryoablation catheter is guided through the pulmonary vein, termed pulmonary vein isolation (PVI), to suppress the aberrant electrical signal to the atria resulting in the atrial fibrillation [22]. The long-term success rate of PVI, including multiple procedures, if necessary, is reported to be 50 to 80%. Andrade et al. [23] and Wazni et al. [24] have both recently reported the superiority of cryoballoon ablation to drug therapy in the prevention of AF recurrence in paroxysmal AF. For patients not felt to be eligible for oral anticoagulants for stroke prevention with persistent AF, left atrial appendage closure is a potential option. In the PROTECT-AF study [25] the Watchman left atrial appendage closure device was found to be noninferior to systemic anticoagulation in the prevention of stroke in patients with AF. Whitlock et al. [26] have reported that left atrial appendage occlusion during cardiac surgery in patients with AF was associated with a lower risk of ischemic stroke or systemic embolism (hazard ratio of 0.67). Of note, most subjects continued to receive antithrombotic therapy during the procedure. Potential approaches to stroke prevention in atrial fibrillation are outlined in Table 3. Other than heighted cardioembolic stroke risk, with roughly a 4-fold increased risk in men and 5.7-fold in women [27], AF can have a deleterious effect on the cerebral circulation and brain function in a less direct way. Persistent AF is associated with reduced cerebral perfusion [28], with resultant reduction in brain volume [29], which can have a deleterious impact on cognitive function The hazard ratio for vascular dementia can be of the order of 1.4 to 1.6 [8,27]. Anxiety and depression are also commonly associated with atrial fibrillation, with one study reporting a prevalence of approximately 35% and “high levels” of depression in roughly 20% [30]. A summary of potential effects of AF on the brain is summarized in Table 4. With NVAF now viewed as being the mechanism in up to one-third of all ischemic strokes [31], the ability to detect paroxysmal AF is of heightened importance. In the PER DIEM trial [32], an implantable electrocardiographic monitoring system for 12 months was compared to prolonged external monitoring for 30 days. The detection rate over 12 months for patients with acute ischemic stroke with no prior detection of AF was 15.3% in those with the implantable device, compared to 4.7% with the external loop recorder. In the Stroke-AF clinical trial, long-term continuous cardiac monitoring with an insertable cardiac monitoring system was compared to what was termed “usual care” external cardiac monitoring after acute ischemic stroke [33]. The detection of AF, lasting more than 30 s over a 12-month period, was significantly higher in the ICM group compared to the control group, 12.1% vs. 1.8%, with a hazard ratio of 7.4, *p* < 0.001. The authors pointed out, however, that this study of enhanced detection rate was not designed to assess the potential effect on outcome.

## 2. The Interrelationship between Cardiac Ischemia and Stroke

Cardiac ischemia can be associated with cardioembolic stroke while massive myocardial infarct (MI) can result in cardiovascular collapse. The latter can be associated with cerebral hypoperfusion with the potential for watershed-type infarction or diffuse cerebral hypoxic–ischemic encephalopathy. Ischemic stroke affects 0.9% of patients with MI within 1 month, and 3.7% within a year after an acute MI. There is a resultant doubled 1-year mortality compared with those not complicated with stroke [34]. Merkler et al. [35] found that, compared to controls without acute MI, the risk of ischemic stroke was highest, roughly 3-fold, during the first 4 weeks after MI (hazard ratio (HR) = 2.7; 95% confidence interval 2.3–3.2). Of note, this risk remained heightened during weeks 5 to 8 (hazard ratio 2.0; 95% confidence interval 1.6–2.4) as well as during weeks 9 to 12 (hazard ratio 1.6; 95% confidence interval 1.3–2.0). This is somewhat in contradistinction to proposed stroke risk that sets a one month timeframe as most pertinent for stroke associated with acute MI [36]. Of particular interest, in terms of longer-term outcome, the one-year mortality was about 15% higher for patients with acute MI plus stroke (51.5%) than for those with MI without stroke (37.1%). Left ventricular (LV) thrombi can develop soon after MI with anterior wall infarction. Such a mechanism is considered the primary cause for MI-associated ischemic stroke. Blood stasis, hypercoagulability and presumably inflammatory components are associated with LV regional wall hypokinesia [37]. Of clinical pertinence, the present of LV thrombi after acute MI has reportedly declined over time. This was as high as 46% in earlier studies [38], but has declined to 15%, in patients with ST-segment-elevation MI (STEMI), with a reported 25% in anterior STEMI [39]. This decline has been attributed to the availability of percutaneous coronary intervention (PCI), antithrombotic agent use, as well as reduced adverse LV remodeling [40].

## 3. The Potential Effects of Heart Failure on the Brain

Heart failure associated with left ventricular (LV) dysfunction is typically seen with an ejection fraction (EF) of ≤40%. This accounts for approximately 50% of all cases of heart failure worldwide [41]. It has been theorized that a clinically significant reduction in EF, associated with progressive LV dilatation and cardiac remodeling, will have a heighted risk for cardioembolic stroke. Of note, AF is associated with a 3-fold increased risk of heart failure in men and 11-fold in women [27]. Isolated congestive heart failure (CHF) appears to be of limited risk, however, in terms of cardioembolism without coexistent factors such as AF. In the Warfarin and Aspirin in Patients with Heart Failure and Sinus Rhythm (WARCEF) trial [42]. Patients with an LVEF of ≤35% randomized to warfarin, with a target international normalized ratio of 2.0 to 3.5, had reduced risk of ischemic stroke (HR = 0.52, *p* = 0.005). However, the composite primary outcome measure of ischemic stroke, intracerebral hemorrhage or death from any cause had a nonsignificant HR of 0.93, *p* = 0.040. This was over a four-year study duration.

The mechanism of embolic stroke is often not readily apparent, and has been termed “embolic stroke of undetermined source” (ESUS), which falls under the category of cryptogenic stroke [43]. Of interest, in a study looking at the relationship between LVEF and wall motion abnormality with ESUS, no relationship was observed [44]. However, the authors did report some relationship between the degree of LVEF reduction and ESUS when they excluded ipsilateral carotid atherosclerosis. This lack of a strong association with LV dysfunction was reinforced in a study of Takatsubo on cardiomyopathy, in which the increased absolute risk of stroke within a year of this stress-related cardiomyopathy was only 0.6% [45]. In the COMMANDER HF trial [46], patients with an EF ≤ 40%, elevated natriuretic peptides and CAD, but in sinus rhythm, were randomized to either rivaroxaban 2.5 mg bid or placebo with continuation of their antiplatelet regimen in both groups. During the median follow-up of 20.5 months, the primary endpoint of all-cause stroke and TIA was reduced by 32% with rivaroxaban with an adjusted HR of 0.68. However, the number needed to treat or to prevent one stroke/TIA was 164 patients per year. Such a study underscores the potential for identifying higher-risk patients for whom anticoagulant therapy is justified. This may be facilitated with the effective use of biomarkers. Measurement of high-sensitivity troponin I, as part of the Atherosclerosis Risk Communities (ARIC) study for example, was assessed in participants aged 54 to 74 years without baseline cardiovascular disease [47]. In an adjusted model, factoring in that 85% of subjects had a detectable level of high-sensitivity troponin I, the highest-level quintile participants had an HR of 2.99 for ischemic stroke compared to the lowest level participants, while the HR for heart failure hospitalization was 4.20. These authors also concluded that high-sensitivity troponin T provided complementary rather than redundant information. Factors associated with increased risk of stroke with heart failure are listed in Table 5.

## 4. Valvular Cardiac Disease and the Brain

Valvular heart disease alone can be associated with an enhanced risk depending upon the severity of the disease and other patient characteristics. In a retrospective Danish study of ischemic stroke comparing subjects with and without aortic stenosis (AS) [48], the HR was 1.31 for all age groups. The HR for younger patients with AS at 18 to 45 years of age was particularly high, at 5.94. This risk markedly dropped in patients older than 65 years who had undergone aortic valve replacement. The coexistence of AF and AS resulted in a 1.5-fold higher risk.

There is an increased risk of ischemic stroke with mitral stenosis (MS). According to one report [49], more than 25% of patients with MS suffer an embolic event without anticoagulation. There is a particularly substantial risk with the combination of rheumatic MS associated with AF, which is a common occurrence. The magnitude of this risk, however, has been called into question, citing an annual risk in nonanticoagulated patients of 5 to 6 per 100 patient years compared to 6 per 100 patient years in the placebo group of NVAF trials [50]. With anticoagulation, however, the risk of stroke or systemic embolism fell into a range of 0.4 to 4 100 patient years [50]. There is generally routine use of vitamin K antagonist (VKA) anticoagulation, with combined rheumatic MS and AF, with a target INR of 2.5 [51]. The use of VKA in patients with rheumatic MS in sinus rhythm is less well-established in terms of risk versus benefit [51]. Of note, this clinical scenario is becoming less frequently encountered according to a Korean study looking at the incidence of MS from 2007 to 2016 [52]. These authors reported that the annual incidence rate of MS declined from 10.3 to 3.6 per 100,000. This correlated with a reduced risk of ischemic stroke, associated with this decline as well as the common use of a VKA. The use of VKA in patients with MS also not unexpectedly correlated with an increased risk of intracerebral hemorrhage. The RE-ALIGN study illustrated the inferiority of a novel oral anticoagulant (NOAC) compared to warfarin in protection against cardioembolic stroke in patients with mechanical heart valves [53]. There were increased rates of both thromboembolism and bleeding complications with the direct thrombin inhibiting agent dabigatran. In addition, non-VK oral anticoagulants have not been studied with rheumatic MS and are therefore not established for efficacy or safety in these patients. For bioprosthetic heart valves, there has been increasing evidence in support of the use of NOACs for patients with coexistent AF [54]. In a study of rivaroxaban compared to warfarin in patients with both AF and a bioprosthetic mitral valve, the NOAC was reported to be noninferior to warfarin in terms of the primary outcome at 12 months of either death, major cardiovascular events or major bleeding [55]. Transcatheter treatment of valvular heart disease is gaining increasing utilization in recognition of enhanced safety [56]. Transcatheter aortic valve implantation (TAVI) can be performed with either a balloon-expandable or self-expanding valve, and is performed percutaneously. It is approved for severe, symptomatic AS. For lower risk patients, the rate of death from any cause, stroke or rehospitalization was 8.5% with TAVI and 15.1% for surgical aortic valve replacement according to one study [57]. For mitral regurgitation, there is now an FDA-approved mitral transcatheter edge-to-edge repair device for higher risk patients with severe symptomatic mitral regurgitation [56]. In the Cardiovascular Outcomes Assessment of the MitraClip Percutaneous Therapy for Heart Failure Patients (COAPT) study [58], intervention resulted in annualized rate of hospital admissions of 35.8% compared to 67.9% for those who received medical therapy alone.

## 5. Stroke Risk and Patent Foramen Ovale (PFO)

A PFO is a common finding in subjects in general with somewhat of a predilection for enhanced ischemic stroke risk. This represents failure of the fetal connection between the right and left atria to close. Although there is typically closure within a few months of birth, a PFO is seen in approximately 25 to 30% of subjects [59]. There are two potential mechanisms in which a PFO can be associated with an increased risk of ischemic stroke. The PFO can serve as a conduit for thromboembolism from a peripheral source, in which thrombus formation, such as in the deep veins of the legs or pelvis, travels from the vena cava to the right atrium and then traverses through the PFO with a right-to-left shunt effect. This places the embolic material to affect the cerebral circulation. Secondly, there could be in situ thrombus formation within the region of the PFO resulting in a cardiogenic source of cerebral embolism.

There are factors which appear to help identify the mechanism of stroke associated with PFO, and this includes younger patients, i.e., those ≤60 years of age, without other identified mechanisms of stroke [60]. The challenge is to identify which patients with cryptogenic stroke who are found to have a PFO and who have the PFO as the most likely mechanism. In a review of 23 case–control studies, the prevalence of PFO in cryptogenic stroke was 40% compared with the commonly cited percentage of 25% in the general population [61]. This is important clinically, as transcatheter closure of the PFO reduces the risk of recurrent stroke in a statistically significant fashion, based upon several studies, with the caveat that the benefit was demonstrated for patients ≤60 years of age who were carefully selected in terms of ruling out other mechanisms [62]. However, this is in recognition that the annualized risk reduction is relatively low, at roughly 0.6%, but that the cumulative risk over time for a relatively young patient can be viewed as substantial. It has been pointed out that TIA and stroke can be seen in older subjects in association with TIA [63,64] and that the pathological mechanism might actually be enhanced in this older population, related to higher incidence of venous thrombosis with age, increased pulmonary pathology which could increase right-sided heart pressure and facilitate a right-to-left shunt as well the potential for an increase in PFO size with age [65]. These authors report on a pooled analysis of four studies of recurrent stroke, in subjects with and without PFO, and identified an enhanced risk of stroke recurrence with PFO only at age ≥65 years with an odds ratio of 2.5. Thus, there is a potential benefit for PFO closure in older patients whose mechanism is most likely related to the PFO. The Risk of Paradoxical Embolism (RoPE) score has been developed to stratify the potential contribution of PFO to embolic stroke of undetermined source. This score incorporates absence of coexistent risk factors for stroke and cortical infarct on imaging and age [66]. In a multicenter study of this score in ESUS [67], this score was validated with a score area under the receiver operating characteristic curve of 0.75. Lower RoPE scores tended to correlate with incidental findings of AF in this study. An issue of particular concern with PFO is the potential risk of ischemic stroke complicating noncardiac surgery. Cumulative information from several studies reports an enhanced risk of up to 16-fold [68]. However, the authors point out that there is a wide range of rates reported, which causes the true risk to be subject to interpretation and the diligence of investigation into the most likely mechanism.

## 6. Stroke Risk and Endocarditis

Endocarditis can be seen in the setting of infection, nonbacterial thrombotic endocarditis and Libman-Sachs endocarditis, typically seen in association with systemic lupus erythematosus and antiphospholipid antibody syndrome [69]. Features which raise concern about infective endocarditis (IE) include susceptible individuals such as those with valvular heart disease with the use of intravenous illicit formulations, along with a stroke presentation associated with fever, heart murmur and leukocytosis. Elevated erythrocyte sedimentation rate is typical, and one can also see positive rheumatoid factor. There can also be associated glomerulonephritis, Roth’s spots and Osler’s nodes. The Duke modified criteria for IE is of clinical utility, and this is in recognition that the clinical feature presentation is often not typical [70]. Major criteria include [1] blood cultures positive for the IE with either typical microorganisms from two separate blood cultures, micoorganisms consistent with IE from persistently positive blood cultures or a single positive blood culture for *Coxiella burnettii,* or an antiphase I IgG antibody titer >1:800 and [2] imaging supportive of endocardial involvement. Blood cultures have a reported sensitivity of up to 90%, but this is dependent on the number of cultures drawn, an extended duration of time of >6 h between drawings and the absence of antibiotic coverage at the time of the blood drawing. TTE has a sensitivity between 50% and 90% and a specificity >90% in the detection of vegetations in native valve endocarditis, with TEE having a sensitivity approaching 100% in native valve endocarditis, and being particularly informative in prosthetic valve endocarditis [51]. Ischemic stroke, associated with septic emboli, is seen in between 20% to 35% of patients depending upon the course of the disease before detection and treatment with up to 50% involvement if one includes clinically silent embolic events [71]. The risk of stroke is relatively high during the first week of presentation and treatment, at 4.8/1000 patient days, and drops precipitously beyond this timeframe [72]. There is concern for mycotic aneurysm formation with secondary intracerebral hemorrhage, which occurs in about 5%, with the majority affecting the middle cerebral artery [73]. A not-uncommon pattern is multiple cortical and subcortical infarcts in different vascular territories with varying degrees of duration [74]. Initiation of antibiotic therapy is of paramount importance, with recognition that *Staphylococcus aureus* now accounts for roughly 30% of cases [75]. There are fairly standardized approaches to assessment and treatment of IE [76]. Antithrombotic therapy is avoided because of concerns about the potential from bleeding associated with antibiotic therapy, and there has been additional concern about the use of thrombolytic therapy [74]. In a systematic review by Bettencourt and Ferro [77], the risk of intracranial hemorrhage was roughly 4.14 times higher in patients treated with thrombolysis and 4.67 times higher for thrombolysis combined with mechanical thrombectomy. However, there was a trend toward improved outcome with thrombectomy alone. Marnat et al. [78] reported that mechanical thrombectomy for ischemic stroke in IE had similar safety and angiographic results to patients presenting with stroke in association with AF. Over the longer term, up to 50% of patients with IE require surgical intervention [75]. Although postponement of cardiac surgical intervention by four weeks is generally recommended by guidelines, there is concern about delay in those with uncontrolled infection [79].

Nonbacterial endocarditis (NBTE) has also been termed “marantic endocarditis”. This is viewed as uncommon and is typically seen as a manifestation of advanced malignancy. It can be associated with ischemic stroke as well as embolic infarction of other organs such as the heart, lungs and gastrointestinal tract. The pathogenesis is reflective of sterile vegetations most commonly affecting the mitral and aortic valves [80]. These vegetations are composed of fibrin and platelet deposits without associated infectious material, and the bacterial cultures are expected to show no growth. In addition to advanced malignancy, as mentioned previously, noninfectious cardiac valvular vegetations can be seen in systemic erythematosus as well as antiphospholipid antibody syndrome [69,81]. In a study of 245 consecutive patients with acute stroke and cancer [82], 20 (8%) were found to have NBTE. In another study of this relationship, 19% of patients with metastatic adenocarcinoma were found to have NBTE [83]. Of pertinence, in this regard, the detection of vegetations will be dependent upon the enhanced detection rate associated with the performance of TEE [84,85]. The treatment of NBTE is based upon effective eradication of the malignancy along with long-term anticoagulation [81].

## 7. Conclusions and Future Directions

The identification of a cardiogenic mechanism of an ischemic stroke has important therapeutic implications with particular reference to anticoagulant therapy. Enhanced cardiac imaging will presumably enhance the diagnostic yield of a cardiogenic source of embolism in cryptogenic stroke. This applies to the emerging applicability of both spectral computed tomography [86] as well as cardiovascular magnetic resonance imaging [87]. Also of potential utility is transcranial Doppler monitoring of the cerebral circulation in the detection of cerebral emboli [88]. Predictive models may also be of value, such as the HAVOC score for predicting AF in patients with cryptogenic cerebral ischemia [89]. Of potential value, in the future, is the application of machine-learning-based statistical algorithms for the identification of cardioembolic stroke derived from electronic health record databases [90].

## Figures and Tables

**Table 1:** Clinical and Radiological Features that Support Cardioembolic Stroke as the Potential Mechanism.

1	An identified cardiogenic source of cerebral embolism;
2	Maximal neurological deficit at the time of onset;
3	Syncope associated with the acute focal neurological deficit;
4	Seizure associated with the onset of the acute neurological deficit;
5	Multiple cerebral vascular territory involvement;
6	Lack of significant extracranial or intracranial occlusive disease other than that associated with the region of acute infarction;
7	Distinct cutoff of the affected vessel, presumably by embolus, as seen on imaging;
8	Cortical involvement in a specific vascular territory such as the superior or inferior division of the middle cerebral artery;
9	Increased risk of spontaneous hemorrhagic transformation of the infarct presumably related to enhanced efforts, by leptomeningeal anastomoses, and other components of the collateral circulation, to re-establish blood flow to the acutely infarcted tissue.

**Table 2:** Stratification of Risk of Stroke and Thromboembolism in Nonvalvular Atrial Fibrillation by the CHA_2_DS_2_-VASc Score.

Risk Factors	Point System
Congestive heart failure/left ventricular	
dysfunction	1
Hypertension	1
Age ≥ 75 years	2
Diabetes mellitus	1
Stroke/Transient ischemic attack	2
Vascular disease (prior myocardial infarct,	
peripheral artery disease)	1
Age 65 to 74 years	1
Female sex	1

**Table 3:** Potential Approaches to Stroke Prevention in Atrial Fibrillation.

1	Oral anticoagulant therapy
2	Electrical cardioversion
3	Antiarrhythmic drug cardioversion
4	Antiarrhythmic drug suppression of atrial fibrillation
5	Cardiac ablation with pulmonary vein isolation (PVI)
6	Left atrial appendage device closure
7	Left atrial appendage surgical closure

**Table 4:** Potential Effects of Atrial Fibrillation on the Brain.

1	Cardioembolic stroke
2	Cerebral hemorrhage as a complication of anticoagulant therapy
3	Cerebral hypoperfusion
4	Vascular Cognitive Impairment and Vascular Dementia
5	Depression

**Table 5:** Potential Factors which may Enhance Cardioembolic Stroke in Heart Failure.

1	Degree of reduction in the left ventricular ejection fraction
2	Associated intracardiac thrombus
3	Coexistent atrial fibrillation
4	Ventricular arrhythmia with resultant cerebral hypoperfusion
5	Elevated natriuretic proteins
6	Elevated high-sensitivity troponin levels

## Data Availability

This was a review article based upon literature review obtained through Pubmed.
